# Incomplete LPS Core-Specific Felix01-Like Virus vB_EcoM_VpaE1

**DOI:** 10.3390/v7122932

**Published:** 2015-11-27

**Authors:** Eugenijus Šimoliūnas, Monika Vilkaitytė, Laura Kaliniene, Aurelija Zajančkauskaitė, Algirdas Kaupinis, Juozas Staniulis, Mindaugas Valius, Rolandas Meškys, Lidija Truncaitė

**Affiliations:** 1 Department of Molecular Microbiology and Biotechnology, Institute of Biochemistry, Vilnius University, Vilnius LT-08662, Lithuania; eugenijus.simoliunas@bchi.vu.lt (E.Š.); m.vilke@gmail.com (M.V.); laura.kaliniene@bchi.vu.lt (L.K.); aurelija.zajanckauskaite@bchi.vu.lt (A.Z.); rolandas.meskys@bchi.vu.lt (R.M.); 2 Proteomics Centre, Institute of Biochemistry, Vilnius University, Vilnius LT-08662, Lithuania; algirdas.kaupinis@gf.vu.lt (A.K.); mindaugas.valius@bchi.vu.lt (M.V.); 3 Laboratory of Plant Viruses, Institute of Botany, Nature Research Centre, Vilnius LT-08412, Lithuania; juozas.staniulis@botanika.lt

**Keywords:** *E. coli* lipopolysaccharide, inner core oligosacharide, bacteriophage vB_EcoM_VpaE1 host-range, genus *Felix01likevirus*, J0101

## Abstract

Bacteriophages represent a valuable source for studying the mechanisms underlying virus-host interactions. A better understanding of the host-specificity of viruses at the molecular level can promote various phage applications, including bacterial diagnostics, antimicrobial therapeutics, and improve methods in molecular biology. In this study, we describe the isolation and characterization of a novel coliphage, vB_EcoM_VpaE1, which has different host specificity than its relatives. Morphology studies, coupled with the results of genomic and proteomic analyses, indicate that vB_EcoM_VpaE1 belongs to the newly proposed genus *Felix01likevirus* in the family *Myoviridae*. The genus *Felix01likevirus* comprises a group of highly similar phages that infect O-antigen-expressing *Salmonella* and *Escherichia coli* (*E. coli*) strains. Phage vB_EcoM_VpaE1 differs from the rest of Felix01-like viruses, since it infects O-antigen-deficient *E. coli* strains with an incomplete core lipopolysaccharide (LPS). We show that vB_EcoM_VpaE1 can infect mutants of *E. coli* that contain various truncations in their LPS, and can even recognize LPS that is truncated down to the inner-core oligosaccharide, showing potential for the control of rough *E. coli* strains, which usually emerge as resistant mutants upon infection by O-Ag-specific phages. Furthermore, VpaE1 can replicate in a wide temperature range from 9 to 49 °C, suggesting that this virus is well adapted to harsh environmental conditions. Since the structural proteins of such phages tend to be rather robust, the receptor-recognizing proteins of VpaE1 are an attractive tool for application in glycan analysis, bacterial diagnostics and antimicrobial therapeutics.

## 1. Introduction

Attachment to cell surface receptors is the first step in phage infection. Phages of Gram-negative bacteria use a variety of cell-associated structures including pili, flagella, outer membrane proteins (Omp) or lipopolysaccharides (LPS) for host cell recognition [1,2,3]. LPS is a major constituent of the bacterial outer membrane (OM). Natural *Escherichia* and *Salmonella* isolates usually contain LPS that consists of: (i) the lipid A, a hydrophobic moiety of the molecule that anchors LPS to the OM; (ii) a phosphorylated, nonrepetitive hetero-oligosaccharide known as the core oligosaccharide (core OS); and (iii) the O-antigen side chain polysaccharide (O-PS or O-Ag) that is a polymer of defined repeat units attached to the core OS, and is usually used for serotyping [4]. The inner part of the core OS is highly conserved within the Gram-negative bacteria, whereas the outer core is more diverse, and the O-Ag is the most variable moiety [5,6].

As mentioned previously, a number of bacteriophages use LPS as a receptor for attachment to the host cell. In turn, as the response to a strong selective pressure imposed by a phage infection, bacteria may evade phage attachment by altering the structure of this complex glycolipid through mutations in genes involved in LPS biosynthesis [7,8,9]. As a result, phage-resistant bacterial strains expressing truncated core LPS can emerge. However, mutation to phage resistance is usually accompanied by pleiotropic fitness costs [10,11]. Indeed, it has been reported that “rough” (O-Ag deficient), and “deep rough” (O-Ag deficient, truncated core OS) LPS mutants are less efficient competitors for growth-limiting resources than strains with intact “smooth” LPS [4,12,13,14]. Although costly in terms of reduced growth rate, such a strategy is often employed by bacteria in response to viral attack during phage treatment. In this regard, while the use of incomplete LPS-specific phages as auxiliary components of phage cocktails has proved beneficial in biocontrol of pathogenic bacteria [15,16,17], it is surprising that only a very limited number of reports on the isolation and characterization of such viruses have been published thus far.

In this study, we describe incomplete core LPS-specific coliphage vB_EcoM_VpaE1 (subsequently, VpaE1) that has been isolated from its natural habitat. Based on the results presented here, VpaE1 belongs to the newly proposed genus *Felix01likevirus*, represented by a well-studied *Salmonella* phage Felix01 [18,19] that infects numerous *Salmonella* strains and has been often used as a diagnostic and therapeutic agent [20,21]. Felix01 has long been considered as a singleton within the family *Myoviridae* [22]. However, during the last few years, a dozen of its close relatives, active mainly against *E.* *coli* [23,24,25,26,27] or *Salmonella* [28,29,30,31,32] have been isolated and sequenced. In addition, more distant Felix01 relatives that infect *Erwinia* [33], *Citrobacter* [34] or *Pseudomonas* [35] have been described. All Felix01-related *Salmonella* or *Escherichia coli* (*E. coli*) phages were isolated on strains with smooth LPS.

Bacteriophage VpaE1 described in this study differs from other characterized Felix01-like viruses by its specificity towards deep rough strains of *E. coli*. By using core LPS mutant strains of *E. coli* K-12 [36], we show that VpaE1 can even use LPS that is truncated down to the inner core OS. Moreover, we demonstrate that VpaE1 can replicate in a wide temperature range from 9 to 49 °C, suggesting that this phage is well adapted to harsh environmental conditions. As a coliphage with unique physiological characteristics, VpaE1 is an attractive model for studying virus-host co-evolution as well as investigating carbohydrate-protein interactions, a research area that has attracted considerable attention in recent years [37]. In addition, to combat the emergence of smooth/rough LPS-specific phage-resistant pathogenic *E. coli* strains, VpaE1 may be used in phage cocktails.

## 2. Materials and Methods

### 2.1. Bacterial Strains and Culture Conditions

Bacterial strains used for host range analysis and phage propagation are listed in Table 1 and Table 2, respectively. Bacteria were cultivated in LB medium, in the temperature range of 7–49 °C. When needed, LB medium was supplemented with either 25 µg·mL^−1^ kanamycin (Kan, Sigma-Aldrich, St. Louis, MO, USA) or 12.5 mg·mL^−1^ tetracycline (Tet, Sigma-Aldrich).

### 2.2. Phage Techniques

Bacteriophage VpaE1 was isolated from avian feces collected at a farm (Vievis, Lithuania) in 2008. Initially, samples were soaked in PB buffer (70 mM NaCl, 10 mM MgSO_4_, 50 mM Na_2_HPO_4_, 30 mM KH_2_PO_4_) and centrifuged at 4000× *g* for 20 min before filtering through 0.45 µm pore-size filters (Sartorius, Goettingen, Germany). Decimal dilutions of the sample were then added to the liquid cultures of *E. coli*, mixed with 2.5 mL of 0.5% (*w*/*v*) soft agar and poured over the 1.2% LB agar plates that were incubated at 37 °C overnight. Then, phage suspensions in LB medium were prepared from single plaques. Decimal dilutions of suspensions were plated on the lawns of *E. coli* B derivatives and incubated in the temperature range of 7 to 49 °C. Thus, phage VpaE1 was selected for further analysis based on its plaque morphology (uniform clear plaques on the lawns of *E. coli* B strains) and a wide temperature range.

Bacteriophage VpaE1 was propagated from a single plaque suspension that was added to the exponentially growing liquid culture of *E. coli* BL21. Then, the culture was incubated at 37 °C with vigorous shaking until complete lysis (~2 h). Phage particles were collected from the lysate by centrifugation at 16,000× *g* for 1 h at 8 °C. The resulting pellet was soaked in 1 mL of SM buffer (100 mM NaCl, 8 mM MgSO_4_, 50 mM Tris-HCl, pH 7.5) containing 0.1 mL CHCl_3_ and DNase I (2 units per mL; Thermo Fisher Scientific, Vilnius, Lithuania) at 4 °C overnight. Next day, the bacterial debris was removed by centrifugation at 5000× *g* for 10 min at room temperature. The supernatant was then subjected to CsCl density gradient centrifugation. The band with the highest opalescence was collected and dialyzed against SM buffer. Purified phage particles were used for TEM, host-range analyses and DNA isolation. Phage stock solution was stored at 4 °C.

The best temperature for VpaE1 propagation was determined by assessing plaque morphology on the lawns of *E. coli* BL21, after the plates were incubated in the temperature range from 9 °C to 49 °C.

### 2.3. Transmission Electron Microscopy (TEM)

Phage suspension was diluted to approximately 10^11^ PFU/mL with distilled water; 5 µL of the sample was deposited directly on the carbon-coated nitrocellulose grid. Excess liquid was drained with filter paper before staining with two successive drops of 2% uranyl acetate (pH 4.5), dried and examined in Morgagni 268(D) transmission electron microscope (Field Electron and Ion Co., Hillsboro, OR, USA). VpaE1 virions were measured (30 virions in total) after calibration with catalase crystals and/or T4 phage particles.

### 2.4. Host Range Analysis

The host range of VpaE1 was investigated using both spot test assay and determination of the efficiency of plating (EOP) as described by Kutter [38].

### 2.5. Adsorption Assays

#### 2.5.1. Assay of Adsorbtion Kinetics

VpaE1 adsorption tests were carried out using *E. coli* BL21 (reference host), *E. coli* BW25113 (VpaE1-resistant negative control), and VpaE1-susceptible mutants of *E. coli* BW25113 from Keio collection [36]. The assay was carried out in triplicate, as described by Kropinski *et al.* [39] with minor modifications. The mid-log-phase cultures of the aforementioned *E. coli* strains (OD_600_ of 0.5) were mixed with phage suspension to give a MOI (multiplicity of infection) of 0.1 and incubated at 37 °C. Samples (0.1 mL) were taken at different time intervals during the time period of 15 min. and diluted with LB medium. Phage-cell complexes were removed by treatment with chloroform (1/10 volume) and centrifugation at 5000× *g* for 5 min. The titer of the unadsorbed phage in the supernatant was then determined by the double-layer agar method.

#### 2.5.2. Periodate and Proteinase K Treatments

To clarify whether the receptor for VpaE1 is a protein or a carbohydrate, VpaE1 adsorption to the proteinase K- or periodate-treated *E. coli* cells was tested by phage pull-down assay essentially as described by Kiljunen *et al.* [40]. For this, cultures of *E. coli* BL21 and BW25113, as well as BW25113 mutants Δ*waag* and Δ*waap* were grown in LB medium at 37 °C with vigorous shaking until mid-log (OD_600_ of 0.5). For proteinase K (Thermo Fisher Scientific) treatment, 2 mL of each culture was collected by centrifugation at 14,000× *g* for 1 min and the pellet was suspended in 2 mL of LB medium. The bacterial suspension was divided into two equal parts: one of these was incubated at 37 °C for 2 h (control sample), while the other was treated with Proteinase K (0.2 mg/mL) at 37 °C for 2 h.

For the periodate treatment, 2 mL of each culture was harvested by centrifugation, as described above. The pellet was then suspended in 1 mL of sodium acetate (50 mM, adjusted to pH 5.2) or 1 mL sodium acetate containing 100 mM of NaIO_4_ (BDH Chemicals Ltd, Poole, England) and incubated at 37 °C for 2 h in the dark.

Treated cells were washed three times with 1 mL of LB medium. After the final wash, the cells were suspended in 1 mL of LB medium. Then, the A_600_ of the bacterial suspension was adjusted to ~0.5, and the phage adsorption assay was carried out. Phage adsorption was assessed by pull-down assay, where centrifugation removes phage particles bound to the bacterial cells. For this, treated bacterial suspensions were incubated with VpaE1 (MOI = 0.1) for 10 min at 37 °C, then cell-phage complexes were removed by centrifugation for 5 min at 5000× *g*, and the number of unadsorbed phage particles remaining in the supernatant was determined in triplicate by the double-layer agar method using *E. coli* BL21. The adsorption ratio was calculated from comparison to a negative control in LB medium without cells.

### 2.6. Phage DNA Isolation and Analysis

Aliquots of phage suspension (10^11^–10^12^ PFU/mL) were subjected to phenol/chloroform extraction and ethanol precipitation. Isolated phage DNA was subsequently used for restriction analysis, or was subjected to genome sequencing. Restriction digestion was performed with Bsu15I, XbaI, HhaI, Csp6I, EcoRV, and CfrI restriction endonucleases (Thermo Fisher Scientific) according to the supplier’s recommendations. DNA fragments were separated by electrophoresis in a 0.8% agarose gel containing ethidium bromide. Restriction analysis was performed in triplicate to confirm the results.

### 2.7. Genome Sequencing, Assembly and Annotation

The complete genome sequence of VpaE1 was determined using Illumina DNA sequencing technology at BaseClear, the Netherlands. Open reading frames (ORFs) were predicted with Glimmer v2.02 [41] and Geneious v5.5.6. [42]. Analysis of the genome sequence was performed using the Fasta-Protein, Fasta-Nucleotide, Fasta-Genome, BLAST2, PSI-Search, Transeq, ClustalW2, (European Bioinformatics Institute, Hinxton, UK), programs, and tRNAscan-SE 1.21 [43] was used to search for tRNAs. The complete genome sequence of vB_EcoM_VpaE1 was deposited in the EMBL nucleotide sequence database under accession number KM657822.

### 2.8. Phylogenetic Analysis

Phylogenetic and molecular evolutionary analyses were conducted using MEGA version 5 [44]. The neighbor-joining tree based on the whole-genome sequence alignment was constructed using Geneious v5.5.6 [42], Genome sequences used for alignment can be found at NCBI: Felix_01 (AF320576); Mushroom (KP143762); FO1a (JF461087); vB_EcoM_AYO145A (KR014248); UAB_Phi87 (JN225449); HY02 (KM092515); O157_phage_12 (KP869110); O157_phage_11 (KP869109); WV8 (EU877232); EC6 (JX560968); JH2 (KF055347); HB-2014 (KP010413); O157_phage_1 (KP869100); phiEa21-4 (FQ482083); and Michonne (KT001916).

### 2.9. Analysis of VpaE1 Virion Structural Proteins

#### 2.9.1. SDS-PAGE

CsCl-purified phage particles (~10^10^) resuspended in a buffer containing 60 mM Tris-HCl (pH 6.8), 1% SDS (*w*/*v*), 1% 2-mercaptoethanol (*v*/*v*), 10% glycerol (*v*/*v*) and 0.01% bromophenol blue (*w/v*) were boiled for 3 min and separated on 12% SDS PAGE following the method described by Laemmli [45]. Protein bands were visualized by staining with PageBlue Protein Staining Solution (Thermo Fisher Scientific).

#### 2.9.2. Filter-Aided Protein Sample Preparation (FASP) for Mass Spectrometry Analysis

CsCl-purified phage particles were concentrated on Amicon Ultra-0.5 mL 30 kDa centrifugal filter unit and were denatured in 8 M urea, 100 mM DTT solution with continuous rotation at 800 rpm in the temperature controlled shaker for 3 hours at 37 °C. Trypsin digestion was performed according to a modified FASP protocol [46,47]. Briefly, phage particles were washed with buffer containing 8 M urea. The proteins were alkylated using iodoacetamide. Buffer was exchanged by washing twice with 50 mM NH_4_HCO_3_, and proteins digested overnight with TPCK Trypsin 20233 (Thermo Scientific, Rockford, IL, USA). Then the peptides were recovered by centrifugation and washed twice with 50% CH_3_CN. Afterwards, the samples were combined, acidified, lyophilized, redissolved in 0.1% formic acid and analyzed by mass spectrometry.

#### 2.9.3. Liquid Chromatography and Mass Spectrometry

Liquid chromatography (LC) separation of trypsin cleaved peptides was performed with a nanoAcquity UPLC system (Waters Corporation, Wilmslow, UK). Peptides were loaded on a reversed-phase trap column PST C18, 100A° 5 μm, 180 µm × 20 mm (Waters Corporation) at a flow-rate of 15 µL/min using loading buffer of 0.1% formic acid and subsequently separated on HSS-T3 C18 1.8 µm, 75 µm × 250 mm analytical column (Waters Corporation) in 60 min linear gradient (A: 0.1% formic acid, B: 100% CH_3_CN and 0.1% formic acid) at a flow rate of 300 nL per min. The analytical column temperature was kept at 40 °C.

The nano-LC was coupled online through a nanoESI 7 cm length, 10 mm tip emitter (New Objective, Woburn, MA, USA) with HDMS Synapt G2 mass spectrometer (Waters Corporation). Data were acquired using Masslynx version 4.1 software (Waters Corporation) in positive ion mode. LC-MS data were collected using data independent acquisition (DIA) mode in combination with online ion mobility separations. The trap and transfer collision energy for high-energy scans in HDMS mode was ramped from 4 to 5 eV and from 27 to 50 eV. For both analyses, the mass range was set to 50–2000 Da with a scan time set to 0.9 s. A reference compound [Glu1]-Fibrinopeptide B (Waters Corporation) was infused continuously (500 fmol/µL at flow rate 500 nL per min) and scanned every 30 s for online mass spectrometer calibration purposes.

#### 2.9.4. Data Processing, Search and Analysis

Raw data files were processed and searched using ProteinLynx Global SERVER (PLGS) version 2.5.3 (Waters Corporation). The following parameters were used to generate peak lists: (i) minimum intensity for precursors was set to 150 counts; (ii) minimum intensity for fragment ions was set to 50 counts; (iii) intensity was set to 500 counts. Processed data was analyzed using trypsin as the cleavage protease, one missed cleavage was allowed and fixed modification was set to carbamidomethylation of cysteines, variable modification was set to oxidation of methionine. Minimum identification criteria included 1 fragment ion per peptide, 3 fragment ions per protein and minimum of 2 peptides per protein. The false discovery rate (FDR) for peptide and protein identification was determined based on the search of a reversed database, which was generated automatically using PLGS when global false discovery rate was set to 4%. The database comprising all possible VpaE1 ORFs (>50 codons) was generated using Geneious v5.5.6 [42], and was used for the identification of VpaE1 structural proteins.

## 3. Results

### 3.1. Phage Isolation and Analysis of VpaE1 Plaque and Virion Morphology

Bacteriophage VpaE1 was isolated in Lithuania while screening for phages with distinct physiological properties [48,49,50,51]. In the course of this screening, no natural bacterial hosts were collected; therefore *E. coli* strain BL21 was used as a reference host for the isolation and propagation of VpaE1.

**Figure 1:**
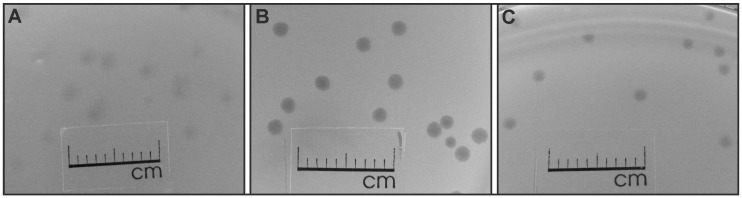
The morphology of VpaE1 plaques produced on the lawns of *E. coli* BL21 at different temperatures. Plaques are shown after: (**A**) five days of incubation at 9 °C; (**B**) 24 h of incubation at 37 °C; and (**C**) 24 h of incubation at 47 °C.

To investigate the effect of temperature on VpaE1 plaque formation, diluted phage suspensions were plated on *E. coli* BL21, and the plates were incubated at 9, 37 and 47 °C. As seen in Figure 1, at 37 °C the plaques produced by VpaE1 (~1.5 mm in diameter) were the most clear and uniform, therefore the subsequent phage propagation and adsorption experiments were carried out at this temperature.

It is worth mentioning that bacteriophage VpaE1 was also capable of multiplying at 49 °C, yet the plaques formed at this temperature were too small to be photographed.

**Figure 2:**
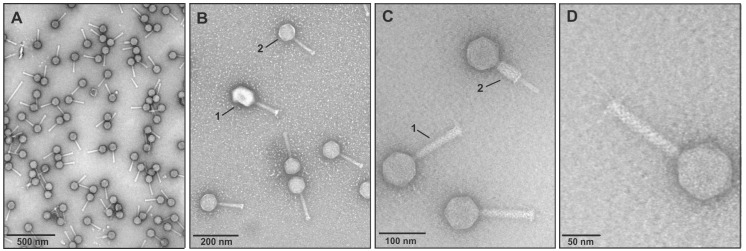
Electron micrographs of bacteriophage VpaE1. (**A** and **B.2**) CsCl density gradient-purified VpaE1 and (**B.1**) T4 particles; (**C**) VpaE1 particles with extended (**C.1**) and contracted (**C.2**) tail; and (**D**) close-up view of VpaE1 particle with tail fibers.

When the content of a single plaque was added to a 10 mL of the exponentially growing liquid culture in LB medium (OD_600_ of 0.5) lysis occurred within ~2 h. At 37 °C, *E. coli* BL21 was capable of producing phage titers of 10^11^–10^12^ pfu mL^−1^. The morphology of CsCl-purified VpaE1 particles was analyzed using transmission electron microscopy (TEM) (Figure 2).

Electron micrographs showed that VpaE1 belongs to the family *Myoviridae* and is characterized by an isometric head of 77 nm in apical diameter and a necked contractile tail of 120 by 16 nm (in the extended form) with long tail fibers connected to the baseplate (Figure 2). In addition, the general appearance of VpaE1 particle is similar to that of other characterized Felix01-like viruses [23,33].

### 3.2. The Host Range of VpaE1

To investigate the host range of VpaE1, a quantitative spot dilution test was employed. As seen in Table 1, those *E. coli* strains that express O-antigen (STEC group) were completely resistant to VpaE1, as were both *Salmonella* and *Klebsiella* strains used in this study. Moreover, the test revealed that *E. coli* K-12 derivatives were also resistant to VpaE1, and only *E. coli* B strains were sensitive to the phage tested.

**Table 1:** Host Range of vB_EcoM_VpaE1.

Strain	Spot Test	Source/Reference
***E. coli*** **B strains**		
B^E^	+	Lindsay W. Black
BL21	+	Novagen
BL21(DE3)	+	Novagen
B40	+	Lindsay W. Black
BE-BS	+	Kenneth N. Kreuzer/[52]
***E. coli*** **K12 strains**		
MG1655	–	Edita Sužiedėlienė
K803	–	Lindsay W. Black
XL1blue	–	Stratagene
CR63	–	Kenneth N. Kreuzer/[52]
BW25113	–	[53]
***E. coli*** **STEC representatives**		
O157:H7	–	Edita Sužiedėlienė
O103:H2	–	Edita Sužiedėlienė
O111:H8	–	Edita Sužiedėlienė
O145:H28	–	Edita Sužiedėlienė
O26:H11	–	Edita Sužiedėlienė
**Other enterobacteria**		
*Salmonella Typhimurium* LT2	–	Jaunius Urbonavičius
*Klebsiella pneumoniae* KV-3	–	[54]

+: clear plaques were observed after incubation at 37 °C; –: no plaques.

### 3.3. Annotation and Overview of VpaE1 Genome

Since many of the genomes of Felix01-related phages have already been examined in detail and published, herein is provided only a brief overview of VpaE1 genome. Bacteriophage VpaE1 has a linear ds DNA genome of 88,403 bp that is similar in size to those of other Felix01-related viruses whose genome sizes range between 84 and 93 kb [19,23,24,27,29,32,33,35]. As is the case with other virulent phages, the VpaE1 genome has an average mol% G+C content of 38.9%, which differs significantly from that of the host (~50%). According to the literature, the drop in G+C content in combination with various forms of DNA modification is known to protect the phage DNA from cleavage by restriction enzymes of the host [55,56].

**Figure 3:**
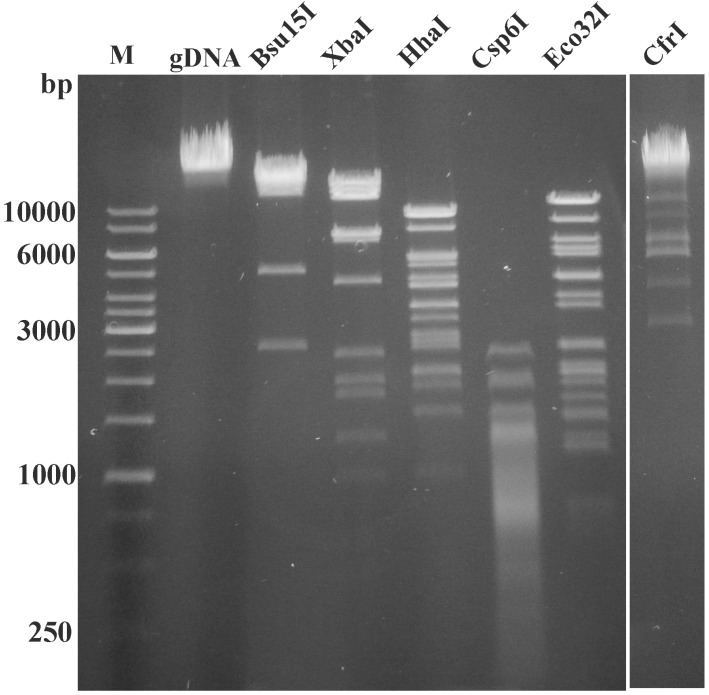
Restriction digestion patterns of phage VpaE1 genomic DNA (gDNA). Lane M represents GeneRuler™ 1 kb DNA Ladder (Thermo Fisher Scientific).

The restriction digestion analysis *in silico* revealed that the DNA of VpaE1 has no recognition sites for many common restriction enzymes (such as BamHI, XhoI, SalI, KpnI, SacI, PstI and even MboI, which recognizes GATC), a feature that is usually observed in phages with unmodified DNA. To test for the digestibility of VpaE1 DNA, 6 restriction enzymes with different sensitivity to DNA methylation were used, namely CfrI (affected by Dcm/CpG methylation), Bsu15I (sensitivity to Dam/CpG methylation), XbaI (sensitivity to Dam methylation), HhaI (affected by CG methylation), Eco32I and Csp6I (both not affected by Dcm, CpG or Dam methylation). Since all enzymes digested the DNA of VpaE1 (Figure 3), restriction analysis indicated that VpaE1 genome contains no modified nucleotides.

**Figure 4:**
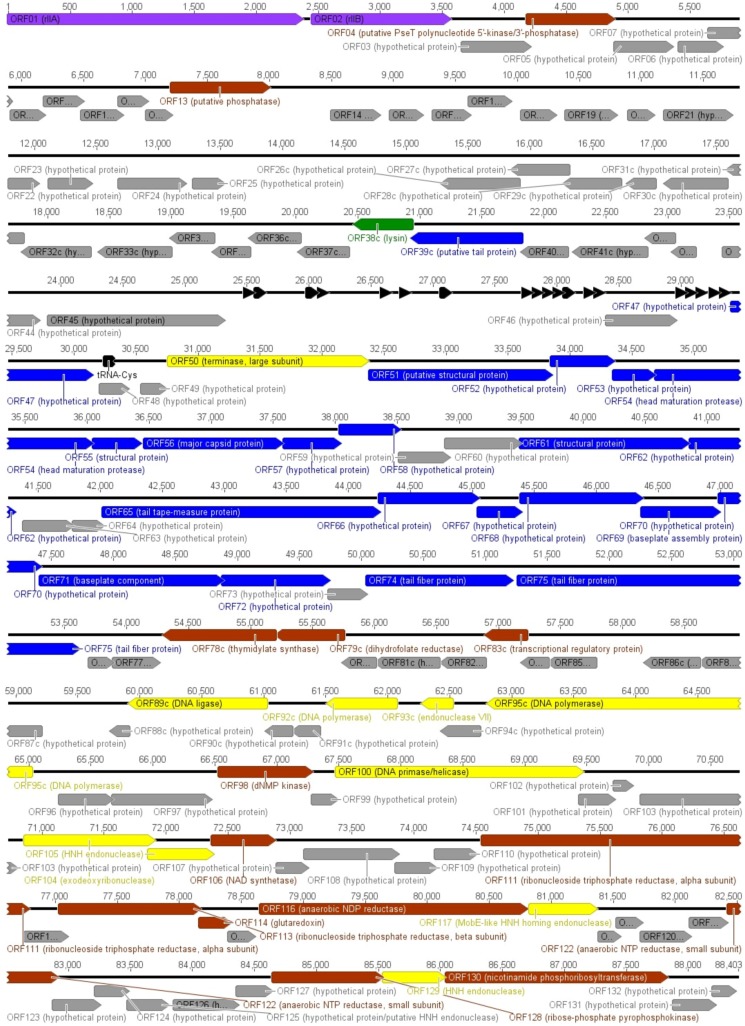
Functional genome map of phage VpaE1. Functions are assigned according to the characterized ORFs in NCBI database and/or MS/MS analysis. The color code is as follows: yellow—DNA replication, recombination, repair and packaging; brown—transcription, translation, nucleotide metabolism; blue—structural proteins; green—lysis; purple—phage-host interaction; and grey—ORFs of unknown function black—tRNA.

This is further supported by the results from bioinformatics analysis, which revealed that no characterized enzymes implicated in DNA modification have homologues in VpaE1. Bioinformatics analysis indicated that the genome of VpaE1 encodes 132 putative open reading frames (ORFs) and 20 tRNAs (Figure 4).

All VpaE1 ORFs had homologues in other sequenced members of *Felix01likevirus* (Table S1). As was the case with other members of *Felix01likevirus*, nearly 70% of VpaE1 ORFs encoded hypothetical proteins with no known functions. Thus, through the examination of homology search results, a putative function could be assigned to only 38 VpaE1 ORFs (Figure 4). In addition, MS/MS analysis allowed for the identification of 11 virion proteins, which were encoded by VpaE1 ORFs with homology to various hypothetical proteins only.

### 3.4. Structural Proteins and Virion Proteome

**Figure 5:**
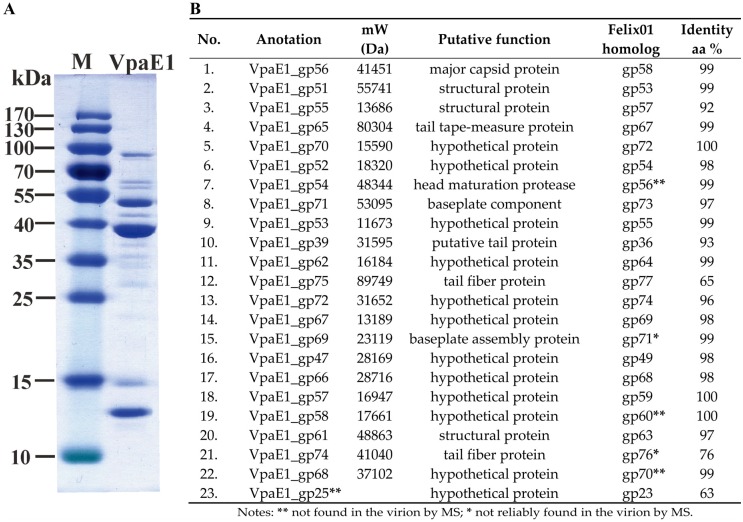
Analysis of protein composition of VpaE1 virion: (**A**) SDS-PAGE analysis of phage VpaE1 particles; and (**B**) the list of VpaE1 structural proteins identified by MS in comparison with those in the virion of Felix01 identified previously [19].

In-gel and in-solution digestion are two common methods used for the analysis of phage structural proteins [57,58]. However, to identify the structural proteins of VpaE1, a recently improved filter-aided sample preparation (FASP) method [46,47] was used. Since following this procedure, most of the contaminants and detergents are being effectively removed from the biological samples. Prior to LC-MS/MS, a quality of phage suspension was verified by SDS PAGE (Figure 5A). Then, reversed-phase nano-liquid chromatography directly coupled with LC-MS/MS analysis was performed that allowed experimental identification of 22 structural proteins of VpaE1 (Figure 5B), including 11 that had no reliable homologues to annotated structural proteins in other organisms.

All identified structural proteins of VpaE1, except the putative tail fiber protein VpaE1_gp75, shared high sequence similarities with their corresponding counterparts in Felix01. Unexpectedly, MS/MS failed to detect a homologue of Felix01_gp23 among the virion proteins of VpaE1. In contrast, three proteins that were detected in VpaE1 (namely, VpaE1_gp54, VpaE1_gp58 and VpaE1_gp68) had not been identified within the particle of Felix01 [19].

### 3.5. Phylogenetic Relatedness

**Figure 6:**
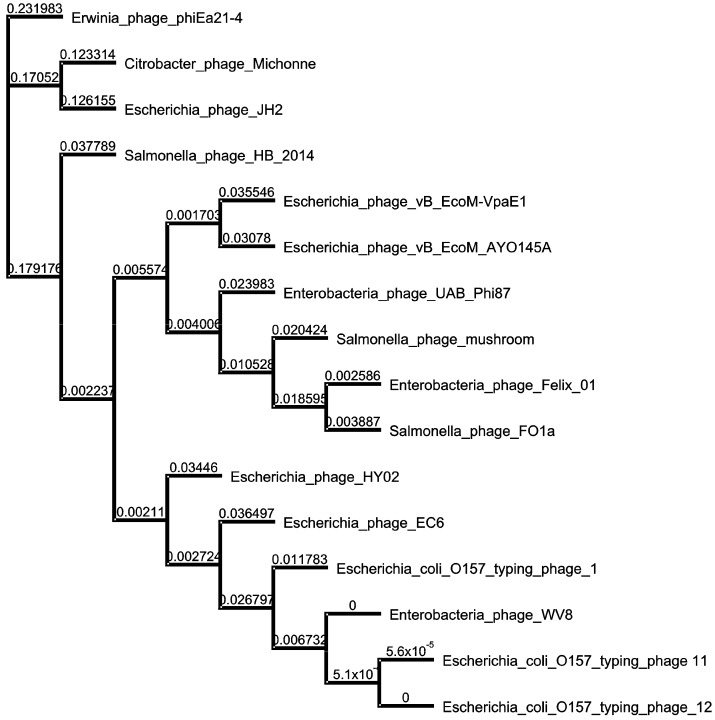
Neighbor-joining tree based on the whole-genome sequence alignment. The numbers at the nodes indicate the bootstrap probabilities.

To assess the evolutionary relationships between VpaE1 and other Felix01-related phages, a comparative whole genome sequence analysis was performed. For this, complete genome sequences of 14 Felix01-like viruses that infect *E. coli* or *Salmonella* strains as well as those of *Erwinia* phage phiEa21-4 and *Citrobacter* phage Michonne were aligned with the genome of VpaE1. As seen in Figure 6, the phylogenetic analysis revealed that Felix01-like viruses form five separate but closely related groups.

Overall, the branching order of groups is somewhat consistent with the defined host range phenotypes seen in *Felix01likevirus*. One such group comprises six *E. coli* O157:H7-infecting phages (namely, WV8, EC6, HY02, ECTP1, ECTP11 and ECTP 12), suggesting that these viruses are likely adapted to avoid specific restriction-modification systems that vary considerably between different lineages of *E.* *coli* [59,60,61]. Bacteriophage JH2, which is the only Felix01-like virus capable of infecting *E. coli* ETEC (enterotoxigenic) serotype O8:H9 [25], together with *Citrobacter* phage Michonne forms a separate divergent branch on the tree. Bacteriophage VpaE1, which is most closely related to *E. coli* O145-serotype-specific phage vB_EcoM_AYO145A isolated in Canada [27], occupies a separate branch together with 5 other Felix01-like viruses that infect *Salmonella* or *Escherichia*.

### 3.6. Identification of VpaE1 Receptor

The results presented in Table 1 suggest that the ability of VpaE1 to adsorb to the host cell is likely influenced by the LPS structure. To confirm this, bacteriophage VpaE1 was tested against a set of *E. coli* K-12 strains that are mutant in the biosynthesis pathway of LPS [36]. Initially, a spotting assay was employed (Table 2).

**Figure 7:**
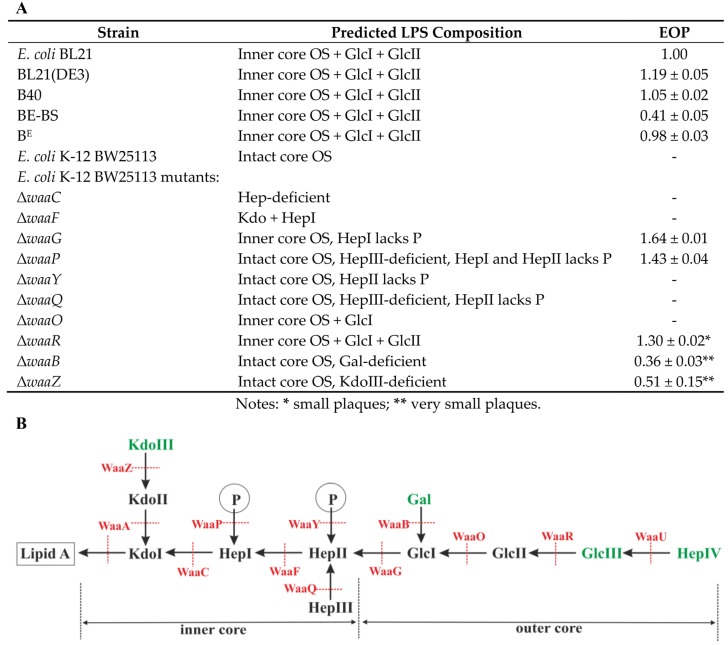
Plating efficiency of VpaE1 on different *E. coli* strains at 37 °C (**A**); and schematic representation of the LPS structure of *E. coli* K-12 (**B**), adapted based on the data from [4,12,13,62,63,64,65,66]. Residues shown in black are shared between *E. coli* K-12 and *E. coli* B LPS.

Then, when the clearly modified infections were observed, determination of the efficiency of plating (EOP) was performed using *E. coli* K-12 BW25113 and other VpaE1-resistant strains from the Keio collection as a negative control (Figure 7A). In the course of this experiment, the ability of VpaE1 to infect different *E. coli* B derivatives was also examined.

**Table 2:** Susceptibility of *E. coli* BW25113 Mutant Strains to VpaE1.

*E. coli* K-12 BW25113 Mutants	Spot Test	Source/Reference
ΔwaaC	–	Keio collection/[36]
ΔwaaF	–	Keio collection/[36]
ΔwaaG	+	Keio collection/[36]
ΔwaaP	+	Keio collection/[36]
ΔwaaY	–	Keio collection/[36]
ΔwaaQ	–	Keio collection/[36]
ΔwaaO	–	Keio collection/[36]
ΔwaaR	+	Keio collection/[36]
ΔwaaB	±	Keio collection/[36]
ΔwaaZ	±	Keio collection/[36]

+: clear plaques were observed after incubation at 37 °C; ±: turbid plaques or no plaques at high dilutions.

As seen in Figure 7, in the case of *E. coli* K-12 BW25113 Δ*waaG*, a significantly higher EOP of VpaE1 was observed than on the lawns of the reference host BL21. The same effect was seen when *E. coli* K-12 BW25113 Δ*waaR* was used, although the plaques formed by VpaE1 on this particular strain were relatively smaller than those on BL21. Therefore, when LPS was truncated even down to the inner core OS, bacteriophage VpaE1 showed higher infectivity towards mutant *E. coli* K-12 strains rather than B derivatives. In addition, the increase in the EOP was also observed when VpaE1 was grown on *E. coli* K-12 Δ*waaP*, a mutant strain with an impaired inner core region (unphosphorylated core, no HepIII added) [12]. Notably, VpaE1 could also infect *E. coli* K Δ*waaB* and Δ*waaZ* (lack Gal and KdoIII residues, respectively), while both Δ*waaF* and Δ*waaC* (the inner core OS truncated down to KdoI and KdoI-HepI, respectively) mutants were resistant to VpaE1 infection. These results suggest that the core residues of the inner core OS are important for VpaE1 adsorption. Unexpectedly, *E. coli* B strains differed in their susceptibility to VpaE1 as well. As seen in Figure 3, the EOP of VpaE1 on *E. coli* BE-BS was reduced more than two-fold, as compared to its EOP on other *E. coli* B-derivatives. However, this puzzling phenomenon needs to be investigated in more depth and is left for future research.

At first glance, it seems that VpaE1 adsorb better when HepI lacks a P moiety, however, our results show that *E. coli* K-12 BW25113 mutants Δ*waaR*, Δ*waaB* and Δ*waaZ* as well as *E. coli* B strains—that all possess phosphorylated HepI—are all susceptible to VpaE1. Therefore, the additional studies are needed to elucidate the precise mechanism of VpaE1 host-cell recognition.

**Figure 8:**
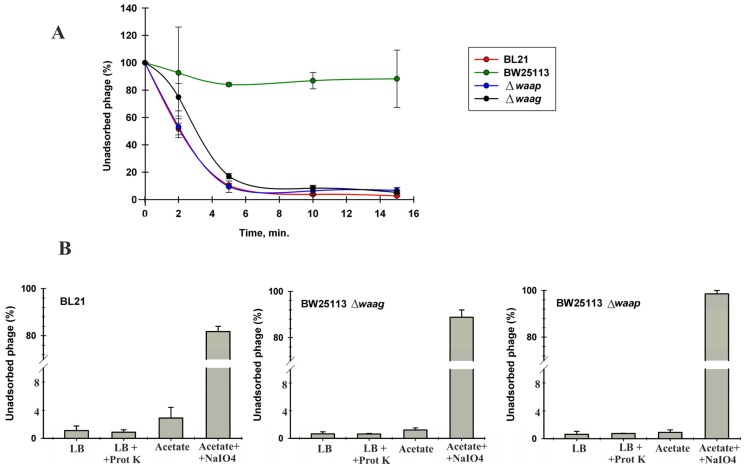
VpaE1 adsorption assays: (**A**) VpaE1 adsorption kinetics; and (**B**) effect of proteinase K, sodium acetate and periodate treatment on the ability of VpaE1 to adsorb to *E. coli* BL21, BW25113 Δ*waaG* and Δ*waaP*.

To confirm that VpaE1 failed to form plaques on *E. coli* BW25113 due to its inability to adsorb to K-12-type LPS, the adsorption assay was performed using several phage-resistant and phage–susceptible strains. The results presented in Figure 8A indicated that bacteriophage VpaE1 indeed failed to adsorb to *E. coli* K-12 BW25113, whereas in the case of *E. coli* BL21, BW25113 Δ*waaG* and Δ*waaP* the adsorption of the phage was unaffected.

To test whether the receptor for VpaE1 is a carbohydrate but not a protein, bacteria were treated with proteinase K or periodate prior to the phage adsorption assay (Figure 8B). Sodium periodate degrades carbohydrates, whereas proteinase K that has a broad substrate specificity degrades cell surface proteins. As seen in Figure 8, the proteinase K treatment had no effect on VpaE1 adsorption, whereas the incubation of *E. coli* cells in the presence of 100 mM periodate reduced VpaE1 adsorption by ~80%. These results indicate that a receptor for bacteriophage VpaE1 is carbohydrate of incomplete core LPS.

## 4. Discussion

Here we present bacteriophage VpaE1 whose virion morphology as well as genome characteristics are reminiscent of those found in the members of the newly proposed genus *Felix01likevirus*, which comprises 14 fully sequenced *Salmonella* and *E. coli* phages. The genomes of Felix01-related phages exhibit a high degree of homology, and VpaE1 is no exception. Genome alignment comparison has shown remarkable similarity (in most cases, >84% nucleotide sequence identity) between the genome of VpaE1 and other sequenced Felix01-like viruses. Moreover, almost all functionally assigned VpaE1 genes, except for gene 75 coding putative tail fiber protein, share >90% amino acid sequence identity with those from the genomes of other Felix01-like viruses, suggesting that the core genome of *Felix01likevirus* is highly conserved. As is the case with Felix01 and its relatives, bacteriophage VpaE1 contains numerous genes involved in DNA replication and metabolism suggesting that Felix01-related viruses, like many other lytic phages, direct their own replication machinery. Also, the genome of VpaE1 codes for 22 apparently functional tRNAs (a feature that has been also documented in other Felix01-like viruses) that may have a positive impact on translation of phage-derived mRNA. Bacteriophage VpaE1 and its relatives do not code for their own RNA polymerase, nor do they code for proteins required for modification of the host RNAP. Hence, in the case of Felix01-like viruses, the question on how transcription is temporally regulated (especially at early stages of infection) remains unanswered.

Despite the aforementioned similarities, bacteriophage VpaE1 profoundly differs from the rest of Felix01-like viruses, since it is the only coliphage among *Felix01likevirus* that does not infect smooth LPS bacteria. All Felix 01-like *Salmonella* and *E. coli* phages characterized thus far have been isolated based on their ability to target a specific serotype of bacteria, and to date, no Felix01-related bacteriophages have been reported to infect O-Ag-deficient strains, much less O-Ag-deficient strains with a truncated core LPS. This could be at least partially due to the fact that such phages seem to be relatively rare in nature, since their rough or deep rough hosts, which usually emerge as a result of the selective pressure imposed by O-Ag/smooth LPS-specific phage infection [3], have been found to be not fit enough to survive the competition for natural resources with smooth strains, and are usually more susceptible to abiotic stress [11,12,13,62]. However short-lived, bacterial populations that produce mutant LPS occasionally do emerge in nature, and so do, apparently, mutant LPS-specific bacteriophages.

In the case of many tailed viruses, the distal part of the phage tail fibers is responsible for host cell recognition. As seen in Figure 5, bacteriophage VpaE1 harbors two putative tail fiber proteins, namely VpaE1_gp74 and VpaE1_gp75. Based on BLASTP analysis, both the C-terminus of VpaE1_gp75 (aa 584–780) and the C-terminal region of VpaE1_gp74 (aa 146–362) show homology to the long tail fiber adhesin of T4 (54% identity/E-value 2e-51 and 37% identity/E-value 9e-32, respectively). In T4, the long tail fiber adhesin is located in the C-terminal domain of gp37, and recognizes *E. coli* B LPS with at least one of the two terminal glucose (Glc) residues present [67] and OmpC found on *E. coli* K-12 but not B derivatives [68]. On the contrary, the host range of VpaE1 is limited to *E. coli* B strains only, since this phage does not use K-12-type LPS as a receptor. Although strikingly similar in genome size and gene content, *E. coli* B and K-12 derivatives mostly differ in the copy number and distribution of IS elements [65,69] that account for the majority of phenotypic differences between the strains [70,71]. According to the literature, *E. coli* B and K-12 derivatives are devoid of the O-antigen since, in both groups, IS elements are inserted at the gene clusters for O-antigen biosynthesis (within *wbbL* in K-12 and *wbbD* in *E. coli* B). However, in the case of *E. coli* B derivatives, due to the IS1 insertion in *waaT*, the core part of LPS is further disrupted leading to the truncated core oligosaccharide [65].

Based on bioinformatics analysis, the tail fiber proteins of VpaE1 and their corresponding counterparts in other Felix01-related phages contain a conserved N-terminus (likely responsible for protein attachment to the phage particle) and a less-conserved C-terminal domain that may be responsible for the attachment of the virus to the host cell. Also, in the case of VpaE1_gp75 and its homologues, the region between the N- and C-terminal domains is highly variable in size and sequence.

**Figure 9:**
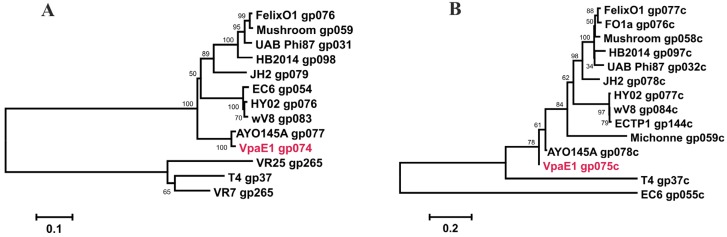
Phylogenetic analysis. Neighbor-joining trees were constructed based on the amino acid sequence alignment of VpaE1_gp74 (**A**) and VpaE1_gp75C (**B**) with tail fiber proteins from various myoviruses. The numbers at the nodes indicate the bootstrap probabilities.

Phylogenetic analysis based on the alignment of the amino acid sequences of putative tail fiber adhesins from various tailed viruses (Figure 9) revealed that the sequences of VpaE1_gp74 and vB_EcoM_AYO145A_gp077 are the most closely related, and both clustered on a separate divergent branch. The same was observed for the sequence of the C-terminal domain (238 aa) of gp75 of phage VpaE1 (VpaE1_gp75C).

As was mentioned above, gp74 and gp75 of VpaE1 both share a region of sequence similarity with gp37 of T4 yet, based on the results of phylogenetic analysis, these three proteins are only distantly related. Nevertheless, considering that both phages recognize *E. coli* B LPS but differ in their ability to use proteins as receptors, the adhesins of T4 and VpaE1 offer a promising model for studying protein structure-function relationship.

The majority of bacteriophages that belong to the newly proposed genus *Felix01likevirus* are highly virulent viruses, and have long been used in diverse applications, such as phage-based identification of bacteria, biocontrol of pathogens, phage-therapy, *etc.* [20,21,28,30,31,72]. Nevertheless, as is the case with antibiotics, the effectiveness of phage applications is greatly limited by the emergence of phage-resistant bacteria that often express rough or deep rough LPS. To surmount this obstacle, the use of phage cocktails that target multiple bacterial receptors has been proposed. In this regard, the isolation of truncated LPS-specific bacteriophages is of great importance.

## 5. Conclusions

Bacteriophage VpaE1 belongs to the newly proposed genus, *Felix01likevirus*, in the family *Myoviridae*. All Felix01-like viruses described to date are highly similar in genome structure and organization, and are adapted to infect O-Ag-expressing *Salmonella* or *Escherichia coli* strains. In contrast, phage VpaE1 presented here is the first Felix01-like virus that infects O-Ag-deficient *E. coli* strains with a truncated core LPS. Moreover, VpaE1 can replicate over a wide temperature range from 9 to 49 °C, suggesting that this phage is well adapted to harsh environmental conditions. Hence, bacteriophage VpaE1 shows potential for the development of phage-based applications.

## Supplementary Materials

The following are available online at http://www.mdpi.com/1999-4915/7/12/2932/s1, Table S1: List of annotated VpaE1 ORFs, Table S2: Data on proteomic analysis of VpaE1 virion proteins.
