# Supplementary material for: Incomplete LPS Core-Specific Felix01-Like Virus vB_EcoM_VpaE1
Source: Viruses. 2015 Nov 27;7(12):6163–81. doi: 10.3390/v7122932 (PMC4690856; doi:10.3390/v7122932)
Supplement: Supplementary file 1 [file viruses-07-02932-s001.pdf]

Supplementary Material: Incomplete LPS Core-specific *Felix01likevirus* vB_EcoM_VpaE1

Eugenijus Šimoliūnas, Monika Vilkaitytė, Laura Kaliniene, Aurelija Zajančkauskaitė, Algirdas Kaupinis, Juozas Staniulis, Mindaugas Valius, Rolandas Meškys and Lidija Truncaitė

Table S1. List of Annotated VpaE1 ORFs.

VpaE1 ORF, Position	Predicted Function (Protein Length aa)	Significant Match (Protein Length aa)	Identity aa %/Similarity aa% (Length of the Overlapping Segment)	E Value
ORF01 1..2367	rIIA (788)	gb|AGF88671.1| rIIA protein *Salmonella* phage FSL SP-010 (786)	98/99 (786)	0.0
ORF02 2449..3558	rIIB (369)	gb|AFU62447.1| hypothetical protein *Escherichia* phage EC6 (369)	99/99 (369)	0.0
ORF03 3660..4208	hypothetical protein (182)	gb|AGF88669.1| hypothetical protein SP010_00055 *Salmonella* phage FSL SP-010 (182)	99/99 (182)	4e-129
ORF04 4186..4881	putative PseT polynucleotide 5'-kinase/3'-phosphatase (231)	ref|NP_944782.1| hypothetical protein Felix01p006 *Salmonella* phage FelixO1 (231)	98/99 (231)	5e-168
ORF05 4892..5356	hypothetical protein (154)	AIK67816.1 hypothetical protein HY02_005 *Escherichia* phage HY02 (159)	97/98 (154)	2e-102
ORF06 5409..5756	hypothetical protein (115)	ref|NP_944790.1| phage conserved protein *Salmonella* phage FelixO1 (115)	100/100 (115)	2e-78
ORF07 5650..5919	hypothetical protein (89)	ref|NP_944791.1| hypothetical protein Felix01p015 *Salmonella* phage FelixO1 (89)	100/100 (89)	2e-57
ORF08 5916..6185	hypothetical protein (89)	gb|AGF89432.1| hypothetical protein SP107_00310 *Salmonella* phage FSL SP-107 (89)	98/100 (89)	3e-59
ORF09 6182..6496	hypothetical protein (104)	ref|NP_944793.2| hypothetical protein Felix01p017 *Salmonella* phage FelixO1 (102)	100/100 (102)	3e-69
ORF10 6182..6496	hypothetical protein (108)	gb|AGR48411.1| hypothetical protein JH2_015 *Escherichia* phage JH2 (108)	96/98 (108)	3e-70
ORF11 6787..7014	hypothetical protein (75)	YP_009146257.1 hypothetical protein HB2014_12 *Salmonella* phage HB-2014 (75)	100/100 (75)	3e-45

Table S1. *Cont.*

VpaE1 ORF, Position	Predicted Function (Protein Length aa)	Significant Match (Protein Length aa)	Identity aa %/Similarity aa% (Length of the Overlapping Segment)	E Value
ORF12 7007..7207	hypothetical protein (66)	ref|YP_002922792.1| hypothetical protein WV8_gp011 *Escherichia* phage wV8 (66)	100/100 (66)	2e-40
ORF13 7209..7994	putative phosphatase (261)	gb|AFQ96092.1| phage conserved protein Enterobacteria phage UAB_Phi87 (261)	98/99 (261)	0.0
ORF14 8504..8884	hypothetical protein (126)	YP_009146262.1 hypothetical protein HB2014_17 *Salmonella* phage HB-2014 (126)	95/98 (126)	4e-82
ORF15 8973..9230	hypothetical protein (85)	AKC04844.1 hypothetical protein AYO145A_016 *Escherichia* phage vB_EcoM_AYO145A (85)	89/94 (85)	9e-45
ORF16 9321..9617	hypothetical protein (98)	AKC04846.1 hypothetical protein AYO145A_018 *Escherichia* phage vB_EcoM_AYO145A (98)	94/97 (98)	2e-60
ORF17 9611..9943	hypothetical protein (110)	gb|AGF88780.1| hypothetical protein SP010_00670 *Salmonella* phage FSL SP-010 (110)	97/98 (110)	8e-69
ORF18 10034..10306	hypothetical protein (90)	ref|NP_944813.1| hypothetical protein Felix01p037 *Salmonella* phage FelixO1 (105)	97/97 (90)	2e-58
ORF19 10393..10797	hypothetical protein (134)	AKC04849.1hypothetical protein AYO145A_021 *Escherichia* phage vB_EcoM_AYO145A (134)	97/97 (134)	5e-89
ORF20 10897..11100	hypothetical protein (67)	YP_009146268.1 hypothetical protein HB2014_23 *Salmonella* phage HB-2014 (67)	97/97 (67)	4e-38
ORF21 11186..11728	hypothetical protein (180)	AIK67833.1 hypothetical protein HY02_022 *Escherichia* phage HY02 (180)	94/97 (180)	2e-121
ORF22 11795..12028	hypothetical protein (77)	gb|AGF88777.1| hypothetical protein SP010_00655 *Salmonella* phage FSL SP-010 (77)	97/100 (77)	1e-47
ORF23 12118..12459	hypothetical protein (113)	gb|AGR48527.1| hypothetical protein JH2_131 *Escherichia* phage JH2 (113)	94/97 (113)	4e-74
ORF24 12682..13212	hypothetical protein (176)	AKC04854.1 hypothetical protein AYO145A_026 *Escherichia* phage vB_EcoM_AYO145A (176)	95/97 (176)	9e-119
ORF25 13281..13529	hypothetical protein (82)	gb|AGF89487.1| hypothetical protein SP107_00590 *Salmonella* phage FSL SP-107 (90)	99/100 (82)	1e-53

Table S1. *Cont.*

VpaE1 ORF, Position	Predicted Function (Protein Length aa)	Significant Match (Protein Length aa)	Identity aa %/Similarity aa% (Length of the Overlapping Segment)	E value
ORF26c 15903..15280	hypothetical protein (207)	gb|AFU63601.1| hypothetical protein *Salmonella* phage SPT-1 (201)	91/97 (196)	2e-130
ORF27c 16306..15842	hypothetical protein (154)	ref|YP_002922810.1| hypothetical protein WV8_gp029 *Escherichia* phage wV8 (154)	97/99 (154)	3e-108
ORF28c 16724..16269	hypothetical protein (151)	gb|AGF88759.1| hypothetical protein SP010_00542 *Salmonella* phage FSL SP-010 (142)	99/100 (142)	8e-100
ORF29c 17003..16779	hypothetical protein (74)	gb|AFU63458.1| hypothetical protein *Salmonella* phage SBA-1781 (74)	100/100 (74)	8e-43
ORF30c 17583..17080	hypothetical protein (167)	AKE44937.1 hypothetical protein ECTP1_00057 *Escherichia coli* O157 typing phage 1 (167)	96/97 (167)	1e-114
ORF31c 17798..17580	hypothetical protein (72)	AKC04862.1 hypothetical protein AYO145A_034 *Escherichia* phage vB_EcoM_AYO145A (77)	100/100 (72)	3e-44
ORF32c 18340..17795	hypothetical protein (181)	ref|NP_944833.1| hypothetical protein Felix01p057 *Salmonella* phage FelixO1 (181)	98/98 (181)	6e-128
ORF33c 18994..18413	hypothetical protein (193)	AKC04863.1 hypothetical protein AYO145A_035 *Escherichia* phage vB_EcoM_AYO145A (193)	98/99 (193)	1e-138
ORF34c 19338..18994	hypothetical protein (114)	ref|YP_002922817.1| hypothetical protein WV8_gp036 *Escherichia* phage wV8 (114)	100/100 (114)	3e-76
ORF35c 19624..19331	hypothetical protein (97)	AKE44932.1 hypothetical protein ECTP1_00052 *Escherichia coli* O157 typing phage 1 (97)	98/100 (97)	9e-63
ORF36c 20031..19624	hypothetical protein (135)	ref|YP_002922819.1| hypothetical protein WV8_gp038 *Escherichia* phage wV8 (129)	93/94 (135)	2e-85
ORF37c 20422..20024	hypothetical protein (132)	YP_009146287.1 hypothetical protein HB2014_42 *Salmonella* phage HB-2014 (132)	95/98 (132)	5e-88
ORF38c 20938..20474	lysin (154)	gb|AGF89474.1| lysin *Salmonella* phage FSL SP-107 (154)	100/100 (154)	2e-109
ORF39c 21822..20938	putative tail protein (294)	gb|AGF88769.1| hypothetical protein SP010_00592 *Salmonella* phage FSL SP-010 (294)	96/96 (294)	0.0

Table S1. *Cont.*

VpaE1 ORF, Position	Predicted Function (Protein Length aa)	Significant Match (Protein Length aa)	Identity aa %/Similarity aa% (Length of the Overlapping Segment)	E Value
ORF40c 22190..21819	hypothetical protein (123)	gb|ACZ55588.1| hypothetical protein *Staphylococcus* phage SA1 (123)	99/99 (123)	3e-85
ORF41c 22828..22238	hypothetical protein (196)	ref|YP_002922824.1| hypothetical protein WV8_gp043 *Escherichia* phage wV8 (196)	99/100 (196)	2e-138
ORF42c 23055..22822	hypothetical protein (77)	ref|YP_002922825.1| hypothetical protein WV8_gp044 *Escherichia* phage wV8 (77)	100/100 (77)	4e-49
ORF43c 23219..23037	hypothetical protein (60)	ref|YP_001504372.1| hypothetical protein Felix01p077 *Salmonella* phage FelixO1 (53)	100/100 (53)	3e-30
ORF44 23448..23819	hypothetical protein (123)	ref|YP_002922827.1| hypothetical protein WV8_gp046 *Escherichia* phage wV8 (123)	100/100 (123)	4e-82
ORF45 23902..25317	hypothetical protein (471)	gb|AGR48505.1| hypothetical protein JH2_109 *Escherichia* phage JH2 (471)	99/99 (471)	0.0
ORF46 28403..28960	hypothetical protein (185)	AKC04878.1 hypothetical protein AYO145A_050 *Escherichia* phage vB_EcoM_AYO145A (185)	99/99 (185)	9e-131
ORF47 29412..30146	hypothetical protein (244)	gb|AFQ96189.1| hypothetical protein Phi87_210 Enterobacteria phage UAB_Phi87 (244)	98/98 (244)	2e-169
ORF48 30209..30436	hypothetical protein (75)	ref|NP_944880.1| hypothetical protein Felix01p101 *Salmonella* phage FelixO1 (75)	97/98 (75)	2e-43
ORF49 30543..30743	hypothetical protein (66)	ref|NP_944882.1| hypothetical protein Felix01p103 *Salmonella* phage FelixO1 (66)	100/100 (66)	1e-37
ORF50 30765..32366	terminase, large subunit (533)	ref|NP_944884.1| terminase, large subunit *Salmonella* phage FelixO1 (533)	100/100 (533)	0.0
ORF51 32383..33849	putative structural protein (488)	ref|YP_002922841.1| hypothetical protein WV8_gp059 *Escherichia* phage wV8 (488)	99/99 (488)	0.0
ORF52 33849..34349	hypothetical protein (166)	gb|AGR48497.1| hypothetical protein JH2_101 *Escherichia* phage JH2 (166)	99/99 (166)	4e-116
ORF53 34349..34681	hypothetical protein (110)	ref|NP_944887.1| hypothetical protein Felix01p108 *Salmonella* phage FelixO1 (110)	99/100 (110)	3e-72

Table S1. *Cont.*

VpaE1 ORF, Position	Predicted Function (Protein Length aa)	Significant Match (Protein Length aa)	Identity aa %/Similarity aa% (Length of the Overlapping Segment)	E Value
ORF54 34693..36039	head maturation protease (448)	ref|NP_944888.1| putative head maturation protease *Salmonella* phage FelixO1 (448)	99/99 (448)	0.0
ORF55 36051..36428	structural protein (125)	AKC04886.1hypothetical protein AYO145A_058 *Escherichia* phage vB_EcoM_AYO145A (125)	97/97 (125)	2e-83
ORF56 36462..37568	major capsid protein (368)	gb|ACZ55521.1| major capsid protein *Staphylococcus* phage SA1 (368)	99/100 (368)	0.0
ORF57 37589..38038	hypothetical protein (149)	ref|NP_944892.1| hypothetical protein Felix01p113 *Salmonella* phage FelixO1 (149)	100/100 (149)	1e-104
ORF58 38038..38520	hypothetical protein (160)	ref|NP_944893.1| phage conserved protein *Salmonella* phage FelixO1 (160)	100/100 (160)	3e-112
ORF59 38517..38918	hypothetical protein (133)	ref|YP_002922850.1| hypothetical protein WV8_gp068 *Escherichia* phage wV8 (133)	99/100 (133)	8e-93
ORF60 38893..39492	hypothetical protein (199)	ref|NP_944895.1| hypothetical protein Felix01p116 *Salmonella* phage FelixO1 (199)	99/99 (199)	6e-142
ORF61 39493..40845	structural protein (450)	gb|AFQ96174.1| phage conserved structural protein Enterobacteria phage UAB_Phi87 (450)	98/99 (450)	0.0
ORF62 40861..41307	hypothetical protein (148)	ref|YP_002922853.1| hypothetical protein WV8_gp071 *Escherichia* phage wV8 (148)	100/100 (148)	3e-104
ORF63 41381..41779	hypothetical protein (132)	ref|NP_944899.1| hypothetical protein Felix01p120 *Salmonella* phage FelixO1 (132)	100/100 (132)	5e-90
ORF64 41782..42021	hypothetical protein (79)	ref|NP_944900.1| hypothetical protein Felix01p121 *Salmonella* phage FelixO1 (79)	100/100 (79)	1e-49
ORF65 42021..44249	tail tape-measure protein (742)	gb|AGF89449.1| hypothetical protein SP107_00395 *Salmonella* phage FSL SP-107 (742)	99/99 (742)	0.0
ORF66 44249..45049	hypothetical protein (266)	gb|AFQ96169.1| hypothetical protein Phi87_180 Enterobacteria phage UAB_Phi87 (266)	99/100 (266)	0.0
ORF67 45049..45390	hypothetical protein (113)	ref|YP_002922858.1| hypothetical protein WV8_gp076 *Escherichia* phage wV8 (113)	100/100 (113)	3e-77

Table S1. *Cont.*

VpaE1 ORF, Position	Predicted Function (Protein Length aa)	Significant Match (Protein Length aa)	Identity aa %/Similarity aa% (Length of the Overlapping Segment)	E Value
ORF68 45390..46367	hypothetical protein (325)	gb|AFU62379.1| hypothetical protein *Escherichia* phage EC6 (325)	99/99 (325)	0.0
ORF69 46367..46990	baseplate assembly protein (207)	gb|AFQ96166.1| hypothetical protein Phi87_175 Enterobacteria phage UAB_Phi87 (207)	100/100 (207)	4e-153
ORF70 46990..47409	hypothetical protein (139)	ref|NP_944914.1| phage conserved protein *Salmonella* phage FelixO1 (139)	100/100 (139)	5e-97
ORF71 47409..48878	baseplate component (489)	gb|AGF89443.1| putative baseplate component *Salmonella* phage FSL SP-107 (489)	98/99 (489)	0.0
ORF72 48881..49738	hypothetical protein (285)	AKC04903.1 hypothetical protein AYO145A_075 *Escherichia* phage vB_EcoM_AYO145A (285)	98/99 (285)	0.0
ORF73 49738..50040	hypothetical protein (100)	gb|AGF89441.1| hypothetical protein SP107_00355 *Salmonella* phage FSL SP-107 (100)	100/100 (100)	3e-66
ORF74 50043..51215	tail fiber protein (390)	AKC04905.1 tail fiber protein *Escherichia* phage vB_EcoM_AYO145A (390)	97/98 (390)	0.0
ORF75 51262..53616	tail fiber protein (784)	gb|AGR48474.1| putative tail fiber protein *Escherichia* phage JH2 (781)	78/84 (788)	0.0
ORF76 53696..53890	hypothetical protein (64)	ref|YP_002922867.1| hypothetical protein WV8_gp085 *Escherichia* phage wV8 (64)	100/100 (64)	1e-36
ORF77 53891..54262	hypothetical protein (123)	gb|AGR48472.1| hypothetical protein JH2_076 *Escherichia* phage JH2 (123)	99/100 (123)	4e-81
ORF78c 55199..54300	thymidylate synthase (299)	gb|AGF88725.1| thymidylate synthase *Salmonella* phage FSL SP-010 (299)	99/99 (299)	0.0
ORF79c 55746..55201	dihydrofolate reductase (181)	ref|YP_002922870.1| dihydrofolate reductase *Escherichia* phage wV8 (181)	98/100 (181)	4e-127
ORF80c 56003..55743	hypothetical protein (88)	ref|YP_002922871.1| hypothetical protein WV8_gp089 *Escherichia* phage wV8 (86)	97/96 (86)	3e-55
ORF81c 56519..56004	hypothetical protein (171)	ref|YP_002922872.1| hypothetical protein WV8_gp090 *Escherichia* phage wV8 (171)	99/100 (171)	6e-119

Table S1. *Cont.*

VpaE1 ORF, Position	Predicted Function (Protein Length aa)	Significant Match (Protein Length aa)	Identity aa %/Similarity aa% (Length of the Overlapping Segment)	E Value
ORF82c 56892..56533	hypothetical protein (119)	gb|AGF88721.1| hypothetical protein SP010_00315 *Salmonella* phage FSL SP-010 (119)	100/100 (119)	5e-82
ORF83c 57223..56894	transcriptional regulatory protein (109)	ref|NP_944934.1| putative transcriptional regulatory protein *Salmonella* phage FelixO1 (109)	99/100 (109)	2e-75
ORF84c 57398..57186	hypothetical protein (70)	ref|YP_002922875.1| hypothetical protein WV8_gp093 *Escherichia* phage wV8 (70)	100/100 (70)	4e-42
ORF85c 57783..57400	hypothetical protein (127)	ref|NP_944938.1| hypothetical protein Felix01p159 *Salmonella* phage FelixO1 (127)	98/98 (127)	1e-86
ORF86c 58615..58175	hypothetical protein (146)	ref|YP_002922877.1| hypothetical protein WV8_gp095 *Escherichia* phage wV8 (146)	100/100 (146)	1e-102
ORF87c 59205..58618	hypothetical protein (195)	ref|YP_002922878.1| hypothetical protein WV8_gp096 *Escherichia* phage wV8 (195)	99/100 (195)	6e-142
ORF88c 59912..59766	hypothetical protein (48)	gb|AFQ96148.1| hypothetical protein Phi87_152 Enterobacteria phage UAB_Phi87 (48)	85/91 (48)	2e-18
ORF89c 61019..59916	DNA ligase (367)	gb|AFU62398.1| ATP dependent DNA ligase *Escherichia* phage EC6 (367)	94/94 (367)	0.0
ORF90c 61222..61016	hypothetical protein (68)	AKC04921.1 hypothetical protein AYO145A_093 *Escherichia* phage vB_EcoM_AYO145A (87)	96/97 (68)	1e-38
ORF91c 61450..61232	hypothetical protein (72)	AKC04924.1 hypothetical protein AYO145A_096 *Escherichia* phage vB_EcoM_AYO145A (72)	97/100 (68)	6e-41
ORF92c 62066..61512	DNA polymerase (184)	gb|AGR48455.1| DNA polymerase *Escherichia* phage JH2 (184)	100/100 (184)	4e-131
ORF93c 62516..62280	endonuclease VII (78)	gb|AFU63489.1| hypothetical protein *Salmonella* phage SBA-1781 (160)	97/98 (78)	9e-48
ORF94c 62740..62435	hypothetical protein (101)	gb|AGR48454.1| hypothetical protein JH2_058 *Escherichia* phage JH2 (160)	99/100 (71)	4e-44
ORF95c 65021..62808	DNA polymerase (737)	gb|AFU63490.1| DNA polymerase *Salmonella* phage SBA-1781 (727)	99/99 (727)	0.0

Table S1. *Cont.*

VpaE1 ORF, Position	Predicted Function (Protein Length aa)	Significant Match (Protein Length aa)	Identity aa %/Similarity aa% (Length of the Overlapping Segment)	E Value
ORF96 65249..65671	hypothetical protein (140)	ref|NP_944959.1| hypothetical protein Felix01p180 *Salmonella* phage FelixO1 (140)	99/99 (140)	1e-96
ORF97 65673..66464	hypothetical protein (263)	gb|AFU63492.1| minor tail protein *Salmonella* phage SBA-1781 (266)	92/96 (263)	2e-170
ORF98 66535..67278	dNMP kinase (247)	AIK67908.1 putative deoxynucleotide monophosphate kinase *Escherichia* phage HY02 (247)	98/98 (247)	5e-177
ORF99 67287..67487	hypothetical protein (66)	ref|NP_944966.1| hypothetical protein Felix01p187 *Salmonella* phage FelixO1 (66)	100/100 (66)	1e-36
ORF100 67480..69465	DNA primase/helicase (661)	YP_009146344.1 putative phage DNA primase/helicase *Salmonella phage* HB-2014 (661)	99/99 (661)	0.0
ORF101 69440..69721	hypothetical protein (93)	ref|YP_002922892.1| hypothetical protein WV8_gp110 *Escherichia* phage wV8 (93)	96/98 (93)	4e-60
ORF102 69718..69867	hypothetical protein (49)	ref|YP_001504375.1| hypothetical protein FelixO1p245 *Salmonella* phage FelixO1 (49)	100/100 (49)	8e-26
ORF103 69940..70797	hypothetical protein (285)	gb|AGF89398.1| hypothetical protein SP107_00140 *Salmonella* phage FSL SP-107 (285)	99/99 (285)	0.0
ORF104 70860..71906	exodeoxyribonuclease (348)	gb|AGF88699.1| putative exodeoxyribonuclease *Salmonella* phage FSL SP-010 (348)	99/99 (348)	0.0
ORF105 71860..72375	HNH endonuclease (171)	gb|AGF88698.1| putative HNH endonuclease *Salmonella* phage FSL SP-010 (163)	99/99 (163)	1e-116
ORF106 72372..72872	NAD synthetase (166)	gb|AGF88697.1| NAD synthetase *Salmonella* phage FSL SP-010 (166)	100/100 (166)	2e-118
ORF107 72894..73142	hypothetical protein (82)	ref|YP_002922898.1| hypothetical protein WV8_gp116 *Escherichia* phage wV8 (82)	100/100 (82)	5e-49
ORF108 73118..73873	hypothetical protein (251)	gb|AGF88695.1| hypothetical protein SP010_00185 *Salmonella* phage FSL SP-010 (251)	99/100 (251)	0.0

Table S1. *Cont.*

VpaE1 ORF, Position	Predicted Function (Protein Length aa)	Significant Match (Protein Length aa)	Identity aa %/Similarity aa% (Length of the Overlapping Segment)	E Value
ORF109 73854..74177	hypothetical protein (107)	ref|NP_944987.2| hypothetical protein Felix01p208 *Salmonella* phage FelixO1 (107)	100/100 (107)	8e-74
ORF110 74170..74502	hypothetical protein (110)	AIK67920.1 hypothetical protein HY02_109 *Escherichia* phage HY02 (110)	98/100 (110)	6e-73
ORF111 74549..76783	ribonucleoside triphosphate reductase, alpha subunit (744)	gb|AGF89405.1| ribonucleoside triphosphate reductase, alpha chain *Salmonella* phage FSL SP-107 (744)	99/99 (744)	0.0
ORF112 76755..77096	hypothetical protein (113)	gb|AFQ96123.1| hypothetical protein Phi87_112 Enterobacteria phage UAB_Phi87 (113)	99/99 (113)	2e-76
ORF113 77033..78166	ribonucleoside triphosphate reductase, beta subunit (377)	ref|NP_944994.1| ribonucleoside triphosphate reductase, beta chain *Salmonella* phage FelixO1 (357)	100/100 (357)	0.0
ORF114 78166..78408	glutaredoxin (80)	gb|AFU63504.1| hypothetical protein *Salmonella* phage SBA-1781 (80)	100/100 (80)	5e-53
ORF115 78401..78607	hypothetical protein (68)	AIK67925.1 hypothetical protein HY02_114 *Escherichia* phage HY02 (68)	100/100 (68)	1e-37
ORF116 78656..80800	anaerobic NDP reductase (714)	gb|ACZ55498.1| anaerobic nucleoside diphosphate reductase *Staphylococcus* phage SA1 (714)	98/99 (714)	0.0
ORF117 80830..81366	MobE-like HNH homing endonuclease (178)	gb|AFU63639.1| hypothetical protein *Salmonella* phage SPT-1 (185)	99/99 (167)	2e-118
ORF118 81388..81555	hypothetical protein (55)	AKC04947.1hypothetical protein AYO145A_119 *Escherichia* phage vB_EcoM_AYO145A (54)	94/96 (54)	6e-27
ORF119 81531..81731	hypothetical protein (66)	gb|AGF89411.1| hypothetical protein SP107_00205 *Salmonella* phage FSL SP-107 (66)	97/96 (66)	1e-37
ORF120 81728..82123	hypothetical protein (131)	ref|NP_945002.1| hypothetical protein Felix01p222 *Salmonella* phage FelixO1 (131)	99/99 (131)	3e-91
ORF121 82120..82419	hypothetical protein (99)	ref|YP_002922911.1| hypothetical protein WV8_gp129 *Escherichia* phage wV8 (99)	98/100 (99)	3e-66

Table S1. *Cont.*

VpaE1 ORF, Position	Predicted Function (Protein Length aa)	Significant Match (Protein Length aa)	Identity aa %/Similarity aa% (Length of the Overlapping Segment)	E Value
ORF122 82429..82914	anaerobic NTP reductase, small subunit (161)	AJF40541.1anaerobic nucleoside-triphosphate reductase subunit *Salmonella* phage Mushroom (161)	99/98 (161)	3e-115
ORF123 82877..83254	hypothetical protein (125)	ref|NP_945005.1| hypothetical protein Felix01p225 *Salmonella* phage FelixO1 (125)	98/99 (125)	3e-82
ORF124 83221..83478	hypothetical protein (85)	ref|NP_945006.1| hypothetical protein Felix01p226 *Salmonella* phage FelixO1 (85)	98/98 (85)	2e-51
ORF125 83481..83801	hypothetical protein (106)	gb|AFU63635.1| hypothetical protein *Salmonella* phage SPT-1 (106)	100/100 (106)	8e-69
ORF126 83853..84368	hypothetical protein (171)	AJF40538.1| hypothetical protein CPT_Mushroom8 *Salmonella* phage Mushroom (171)	99/99 (171)	2e-123
ORF127 84361..84639	hypothetical protein (92)	gb|AFQ96109.1| hypothetical protein Phi87_92 Enterobacteria phage UAB_Phi87 (92)	98/100 (92)	4e-61
ORF128 84651..85532	ribose-phosphate pyrophosphokinase (293)	gb|AFU63632.1| hypothetical protein *Salmonella* phage SPT-1 (293)	98/98 (293)	0.0
ORF129 85541..86032	HNH endonuclease (163)	gb|AFU63402.1| hypothetical protein *Salmonella* phage SBA-1781 (163)	100/100 (163)	1e-116
ORF130 86047..87828	nicotinamide phosphoribosyltransferase (593)	ref|NP_945019.1| Putative nictotinate phosphoribosyltransferase *Salmonella* phage FelixO1 (593)	98/99 (593)	0.0
ORF131 87882..88217	hypothetical protein (111)	gb|AGF88673.1| hypothetical protein SP010_00075 *Salmonella* phage FSL SP-010 (111)	98/100 (111)	2e-74
ORF132 88199..88375	hypothetical protein (58)	ref|NP_945023.1| hypothetical membrane protein *Salmonella* phage FelixO1 (58)	100/100 (58)	2e-34

© 2015 by the authors; licensee MDPI, Basel, Switzerland. This article is an open access article distributed under the terms and conditions of the Creative Commons by Attribution (CC-BY) license (http://creativecommons.org/licenses/by/4.0/).
